# Hybrid-Actuated Multimodal Cephalopod-Inspired Underwater Robot

**DOI:** 10.3390/biomimetics11010029

**Published:** 2026-01-02

**Authors:** Zeyu Jian, Qinlin Han, Tongfu He, Chen Chang, Shihang Long, Gaoming Liang, Ziang Xu, Yuhan Xian, Xiaohan Guo

**Affiliations:** 1 College of Engineering, Ocean University of China, Qingdao 266100, China; 2 State Key Laboratory of Robotics and Intelligent Systems, Shenyang Institute of Automation, Chinese Academy of Sciences, Shenyang 110016, China; 3 University of Chinese Academy of Sciences, Beijing 100049, China; 4 Academic Affairs Office, Ocean University of China, Qingdao 266100, China

**Keywords:** underwater robot, bio-inspired robot, hybrid propulsion, undulatory fin, flapping tentacle

## Abstract

To overcome the limitations in maneuverability and adaptability of traditional underwater vehicles, a novel hybrid-actuated, multimodal cephalopod-inspired robot is proposed. This robot innovatively integrates a hybrid drive system wherein sinusoidal undulating fins provide primary propulsion and steering, water-flapping tentacles offer auxiliary burst propulsion, and a gear-and-rack center-of-gravity (CoG) adjustment module modulates the pitch angle to enable depth control through hydrodynamic lift during forward motion. The effectiveness of the design was validated through a series of experiments. Thrust tests demonstrated that the undulating fin thrust scales quadratically with oscillation frequency, aligning with hydrodynamic theory. Mobility experiments confirmed the multi-degree-of-freedom control of the robot, demonstrating effective diving and surfacing via the CoG module and high maneuverability, achieving a turning radius of approximately 15 cm through differential fin control. Furthermore, field trials in an outdoor artificial lake with a depth of less than 1 m validated its environmental robustness. These results confirm the versatile maneuvering capabilities of the robot and its robust adaptability to confined and shallow-water environments, presenting a novel platform for complex underwater observation tasks.

## 1. Introduction

Through millions of years of natural selection, aquatic organisms have developed highly optimized morphological architectures that confer exceptional hydrodynamic efficiency. In particular, fish exhibit advanced bioengineering strategies, employing flexible axial skeletons for undulatory locomotion to achieve agile movement in complex aquatic environments. Investigations into piscine locomotion mechanisms have significantly influenced the field of biomimetics [1], while also motivating the design of novel bio-inspired autonomous underwater vehicles.

In bio-inspired fish research, the median and paired fin locomotion mode has garnered significant attention due to its high maneuverability [2]. One major branch focuses on imitating the undulation of the wide pectoral fins of stingrays. Inspired by this, researchers have developed robots demonstrating excellent short-distance hovering capabilities [3,4]. Some studies have further utilized flexible materials to fabricate biomimetic pectoral fins, aiming to achieve a fine balance between speed and maneuverability [5].

Another line of research focuses on the long, ribbon-like fins of species like Gymnarchus niloticus and knifefish. These fins enable high-precision forward, backward, and turning motions [6]. Key to this superior maneuverability is the ability of the fin to generate counter-propagating waves, where two waves travel from opposite ends of the fin and meet at a controllable nodal point to achieve hovering [7,8].

To deeply understand these propulsive mechanisms, extensive work has been done on modeling and analysis. For example, parametric kinematic models have been proposed for fin-surface motion [9,10,11]. Researchers have also combined Computational Fluid Dynamics with dynamic mathematical equations to optimize structural configurations [12,13]. Modern numerical studies further analyze the scaling laws between kinematic parameters and hydrodynamic performance [14]. Concurrently, experimental techniques like Particle Image Velocimetry are widely used to validate these numerical models and visualize the three-dimensional flow fields around the fins [15,16], revealing complex wake structures like oblique jets and vortex tubes [17].

Although undulatory fins alone can achieve bio-inspired locomotion, cephalopods like squids and cuttlefish employ a multimodal propulsion strategy. While pulsed-jet propulsion is the primary mechanism for high-speed bursts and has been successfully replicated in robots for impulsive acceleration [18,19], they utilize a combination of undulatory fins and flapping tentacles for cruising and maneuvering. This study focuses on mimicking this hybrid fin-tentacle actuation strategy to enhance robotic maneuverability. This integrative strategy not only enhances maneuverability [20] but also partially improves propulsive efficiency.

This study introduces an underwater bio-inspired robot whose propulsion strategy replaces conventional propellers with a combination of sinusoidal undulating fins and water-flapping tentacles. Dynamic depth control is accomplished via a pitch control mechanism, achieved through center-of-gravity (CoG) adjustments, which facilitates stable ascent and descent. The robot features advantages such as high bio-friendliness and excellent maneuverability, making it highly suitable for a variety of underwater observation tasks.

In this paper, we make the following key contributions: (1) We designed a water-flapping tentacle integrating an automatic resistance adjustment mechanism to reduce drag and a torsion spring structure to improve terrain adaptability in confined spaces. (2) We developed a system where the sinusoidal undulating fins achieve propulsion and steering, while a separate CoG adjustment module controls pitch, collaboratively enabling flexible spatial maneuverability. By coupling the pitch angle adjustment with forward propulsion, the robot can effectively execute three-dimensional maneuvers, including diving and surfacing. (3) We built an experimental robotic prototype to verify the performance of both bionic propulsion structures and confirm the feasibility of its dual motion modes through pool tests and outdoor trials.

## 2. Overall Design of the Robot

### 2.1. Main Components of the Robot

**Figure 1:**
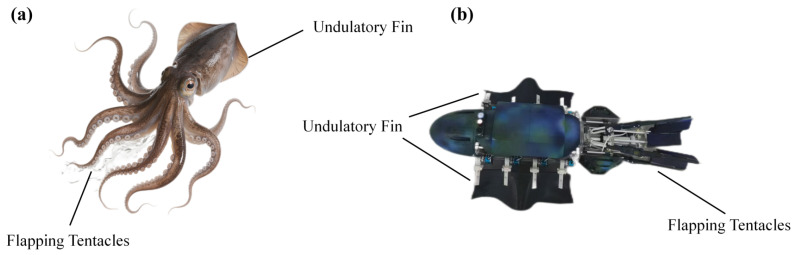
Comparison between real-world squids and bionic robots: (**a**) Real squids. (**b**) Physical prototype.

In nature, cephalopods like squids are renowned for their versatile swimming capabilities. While they rely on pulsed-jet propulsion for rapid escape, their cruising and precise maneuvering are often achieved through the coordination of fins and tentacles. Through bionic observations, we have focused on this specific integration of multiple swimming modes. Inspired by this multimodal propulsion mechanism, we have developed a bionic robot with two swimming modes, as illustrated in Figure 1.

**Figure 2:**
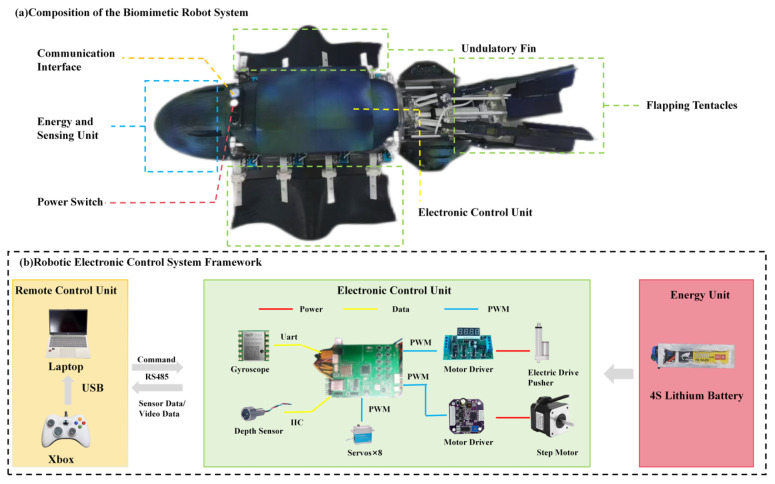
(**a**) Composition of the biomimetic robot system. (**b**) Robotic electronic control system framework.

As illustrated in Figure 2a, the robot employs a modular design, with the main body segmented into three components: a head energy and sensing module, a central sinusoidal undulating fin section, and the water-flapping tentacles unit. Each module is independently sealed, enabling easy disassembly and replacement at any time. The head module houses a camera and a lithium battery. The middle section contains an internal main control compartment, a pair of lateral undulatory fins, and a bottom-mounted CoG adjustment module. The tail is composed of National Advisory Committee for Aeronautics (NACA) airfoil-shaped water-flapping tentacles for propulsion.

Figure 2b illustrates the electronic control system framework, which is organized into three primary sections. The remote control unit serves as the human–machine interface, utilizing a laptop and an Xbox controller. It sends commands and receives sensor signals via an RS485 connection. The energy unit consists of a 4S lithium battery that supplies power to the system. The electronic control unit acts as the brain of the robot. It receives power from the energy unit and processes data from the attitude and environmental sensors (a gyroscope and a depth sensor). The central MCU then sends pulse width modulation command signals to the various drivers. These include motor drivers that control the eight servos for the undulating fins and the stepper motor for the CoG adjustment module, as well as an electric drive pusher for the flapping tentacles.

The detailed specifications of the robot are summarized in Table 1.

### 2.2. Design of Undulatory Fin

**Figure 3:**
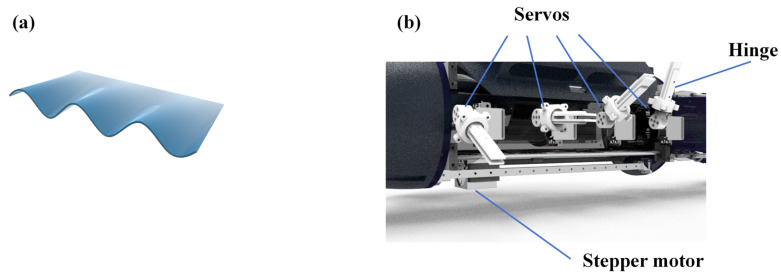
(**a**) The shape of the fin surface. (**b**) Mechanical structure of the undulating fin.

As shown in Figure 3a, the design of the undulating fin is inspired by natural organisms, in which the fin surfaces are discretized to mimic biological undulation. The mechanical structure is detailed in Figure 3b. Each fin surface is controlled by four servo motors. In addition, hinge connections are used in the design of the fin rays, which can ensure that transverse waves are generated during the movement of the undulating fin.

To achieve surfacing and diving, the robot employs a CoG adjustment mechanism, which is also integrated into the mechanical structure shown in Figure 3b. Considering the disadvantages of traditional screw-type center of gravity adjustment mechanisms, such as insensitive movement, slow adjustment speed, and high weight, this robot utilizes a gear-and-rack CoG adjustment mechanism. This mechanism comprises a stepper motor with its bracket, a gear–rack pair, and a rail slider. The rack is fixed on the robot, the stepper motor bracket fixes the stepper motor on the slider, and the gear is mounted on the stepper motor and meshes with the rack. During operation, the rack remains stationary while the gear rolls along it, thereby driving the motor assembly to slide and realizing CoG adjustment. In this manner, the robot achieves ascent and descent by modulating its pitch angle.

### 2.3. Design of Water-Flapping Tentacles

**Figure 4:**
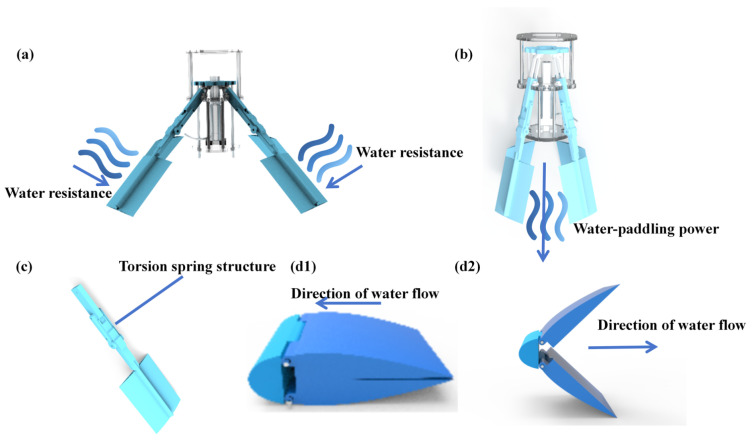
Working schematic diagram of the water-flapping tentacle: (**a**) The water-flapping tentacles are opened. (**b**) The water-flapping tentacles are beating the water. (**c**) Structure of a single water-flapping tentacle with a torsion spring. (**d1**) The water-facing state of the tentacles. (**d2**) The water-flapping state of the tentacles.

To achieve high-speed locomotion, the robot is equipped with a propulsion system driven by an electric drive pusher. As shown in Figure 4a,b, a single motor simultaneously drives the tentacles to perform flapping motions. During the return stroke, the pushrod moves the chassis backward, which in turn drives the swing rod and tentacles to swing forward. Conversely, during the power stroke, the pushrod moves the chassis forward, causing the swing rod and tentacles to swing backward. Propulsion is generated during the backward swing of the tentacles.

As shown in Figure 4c, to enhance the terrain adaptability of the robot, torsion springs have been incorporated into the water-flapping tentacles. When operating in confined spaces, the tentacles experience external forces that cause them to bend. In this process, the torsion spring within the joint twists, causing the joint to bend inward and the entire tentacle to assume a curved configuration.

To minimize hydrodynamic resistance, the robot is equipped with tentacles capable of automatic resistance adjustment. As shown in Figure 4(d1), the tentacles passively close during the return stroke. During the power stroke, they passively open to maximize thrust, as shown in Figure 4(d2). This mechanism effectively balances thrust generation and hydrodynamic resistance. Furthermore, the tentacle surface design is inspired by the wings of underwater gliders. By adopting the NACA airfoil, the resistance during movement is reduced.

## 3. Mathematical Modeling and Analysis of Bionic Propulsors

### 3.1. Hydrodynamics of Underwater Propulsion

To analyze the hydrodynamic characteristics of the undulating fin, Lighthill’s elongated-body theory [21,22] provides a framework for calculating the reactive forces acting on a swimming body. These forces originate from the necessary acceleration of an added mass of water by the body’s undulations. The theory simplifies the complex 3D problem by treating the elongated body as a series of independent 2D slices along its length, allowing for the calculation of net thrust.

**Figure 5:**
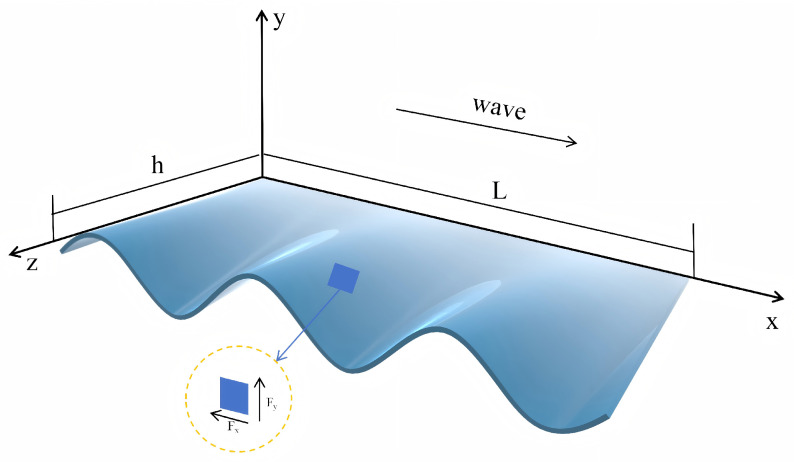
The undulating fin in the coordinate system.

As shown in Figure 5, we define a coordinate system where the body axis is along the *x*-direction, transverse undulation occurs in the *y*-direction, and the fin width is along the *z*-direction. The traveling wave profile of the fin, which has length *L*, can then be expressed as

(1) y(x,t)=A(x)sin(kx−ωt)

where A(x) is the amplitude profile along the fin, *k* is the wave number, and ω is the angular frequency. The instantaneous transverse velocity of the fin at any position *x* can be expressed as

(2) $$v_{y}\left(x,t\right)=\frac{\partial y}{\partial t}=-A\left(x\right)\omega \cos\left(kx-\omega t\right)$$

As the fin moves, each 2D slice displaces the surrounding fluid. The added mass per unit length $m_{a}$ for a slice with local width h(x) can be modeled based on potential flow theory. For an equivalent elliptical or flat-plate cross-section, this is given by

(3) $$m_{a}\left(x\right)=C_{m}\rho h{\left(x\right)}^{2}$$

where h(x) is the local width of the fin, ρ is the fluid density, and $C_{m}$ is a non-dimensional added mass coefficient. The transverse reactive force per unit length $F_{y}$ is the product of this added mass and its acceleration:

(4) $$F_{y}\left(x,t\right)=-m_{a}\left(x\right)\frac{\partial v_{y}}{\partial t}=-m_{a}\left(x\right)\left(-A\left(x\right)\omega^{2}\sin\left(kx-\omega t\right)\right)$$

Since the fin surface is at an angle to the direction of travel, this lateral force $F_{y}$ creates a forward-directed thrust component. By integrating this force component along the fin length *L* and averaging over a full oscillation cycle, the mean thrust *T* can be determined. This resulting thrust is a function of the fluid density, fin dimensions, and kinematics. Using characteristic values for the fin width *h* and amplitude *A*, and the relationship ω=2πf, the mean thrust is expressed as

(5) $$T=C_{T}\rho kLh^{2}A^{2}f^{2}$$

where $C_{T}$ is a non-dimensional thrust coefficient. It is important to note that Lighthill’s theory was originally developed for slender bodies. Applying it to paired pectoral fins is a quasi-steady simplification where complex three-dimensional hydrodynamics are lumped into the empirical coefficient $C_{T}$. While more rigorous dynamic modeling of flexible links in fluid environments can be achieved using methods such as the Gibbs-Appell formulation [23,24], the simplified Lighthill model is adopted here to provide a computationally efficient scaling law suitable for real-time control.

According to Equation (5), the mean thrust generated by the undulating fin scales quadratically with the oscillation frequency *f*. This relationship provides a valuable theoretical basis for motion control and parameter optimization of undulating fin propulsion systems.

### 3.2. Water-Flapping Tentacles

**Figure 6:**
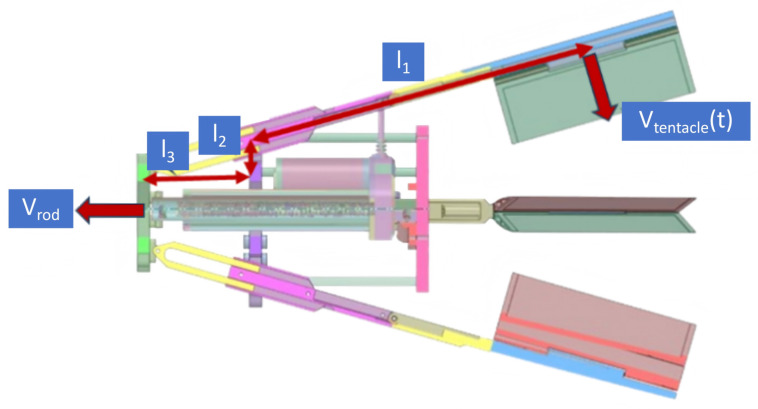
The model of water-flapping tentacles.

For the dynamic modeling and analysis of the water-flapping tentacles, this paper adopts a quasi-steady model based on modified blade-element momentum principles, which is better suited for unsteady flapping propulsion [25]. This approach calculates the thrust based on the instantaneous momentum imparted to the fluid by the tentacle. As illustrated in Figure 6, the tail thruster drives the water-flapping tentacles via the linear motion of the pushrod, which moves at velocity $V_{\mathrm{rod}}$. This motion is converted into the flapping of the tentacles by a linkage mechanism. The instantaneous flapping velocity $V_{\mathrm{tentacle}}$ is determined by the kinematic relationship of the linkage mechanism. As shown in Figure 6, the key geometric parameters are defined as the swing arm length $l_{1}$, the coupler length $l_{2}$, and the instantaneous horizontal displacement $l_{3}$. The linear motion of the pushrod $V_{\mathrm{rod}}$ is converted into the angular rotation of the swing arm.

Based on the linkage geometry, the tangential velocity of the tentacle tip $V_{\mathrm{tentacle}}$ relates to the pushrod velocity through a geometric transmission ratio J(θ):

(6) $$V_{\mathrm{tentacle}}\left(t\right)=J\left(\theta \right)\cdot V_{\mathrm{rod}}\left(t\right)$$

where J(θ) is the instantaneous Jacobian determined by the linkage geometry $l_{1},\;l_{2},\;l_{3}$ and the current angle θ.

To determine the required driving force of the linear actuator $F_{\mathrm{actuator}}$, we apply the principle of virtual work. The single actuator simultaneously drives *N* tentacles (where N=4 in this prototype). Therefore, the actuator must overcome the total hydrodynamic drag torque. The input power from the actuator equals the total output power dissipated by the hydrodynamic load:

(7) $$F_{\mathrm{actuator}}\cdot V_{\mathrm{rod}}=N\cdot \left(F_{\mathrm{drag}}\cdot V_{\mathrm{tentacle}}\right)$$

Substituting the kinematic relationship from Equation (6) and the hydrodynamic drag model $F_{\mathrm{drag}}=\frac{1}{2}\rho C_{D}S_{\mathrm{tentacle}}V_{\mathrm{tentacle}}^{2}$, the required actuator force is derived as follows:

(8) $$F_{\mathrm{actuator}}=N\cdot \left(\frac{1}{2}\rho C_{D}S_{\mathrm{tentacle}}{\left(V_{\mathrm{tentacle}}\right)}^{2}\right)\cdot J\left(\theta \right)$$

This equation quantifies the total load on the linear motor, demonstrating that the required force scales with the number of tentacles, the square of the velocity, and the instantaneous transmission ratio.

As modeled, the effective area of the water-flapping tentacle $S_{\mathrm{tentacle}}$ is not constant. Based on the automatic resistance adjustment design, the tentacles passively open during the power stroke and close during the return stroke. This transient area change can be approximated by a first-order response model

(9) $$S_{\mathrm{tentacle}}\left(t\right)=\left\{\begin{array}{cc} A_{\max}\left(1-e^{-t/\tau_{\mathrm{open}}}\right) & \left(\mathrm{Power}\;\mathrm{Stroke}\right)\\ A_{\min}+\left(A_{\max}-A_{\min}\right)e^{-t/\tau_{\mathrm{close}}} & \left(\mathrm{Return}\;\mathrm{Stroke}\right) \end{array}\right.$$

where $A_{\max}$ and $A_{\min}$ are the maximum and minimum effective areas, respectively, *t* is the time elapsed since the beginning of the stroke, and $\tau_{\mathrm{open}}$ and $\tau_{\mathrm{close}}$ are the time constants for the passive opening and closing actions.

A key advantage of this design is the automatic resistance adjustment. Unlike a simple fixed-area tentacle, the adaptive tentacle passively closes during the return stroke. This drastically reduces the effective area $S_{\mathrm{tentacle}}$ during the return phase.

According to the blade-element momentum model, the local fluid velocity at the tentacle surface $V_{\mathrm{fluid}}$ is modeled as a function of the freestream velocity and an induced velocity component

(10) $$V_{\mathrm{fluid}}\left(t\right)=U+k_{v2}\left[V_{\mathrm{tentacle}}\left(t\right)-U\right]$$

where *U* is the forward speed of the robot, also known as the freestream velocity, $k_{v2}$ is the axial induction factor, which has a value between 0 and 1, and $V_{\mathrm{tentacle}}\left(t\right)$ is the instantaneous flapping velocity.

Then, the instantaneous thrust force $F_{\mathrm{thrust}}$ generated during the backward-flapping power stroke is calculated from the momentum flux equation

(11) $$F_{\mathrm{thrust}}\left(t\right)=\rho S_{\mathrm{power}}\left(t\right)\cdot V_{\mathrm{fluid}}\left(t\right)\cdot \left(V_{\mathrm{tentacle}}\left(t\right)-U\right)$$

where $S_{\mathrm{power}}$ is the effective area during the power stroke as given by Equation (9), and $V_{\mathrm{fluid}}$ is the local fluid velocity from Equation (10).

**Figure 7:**
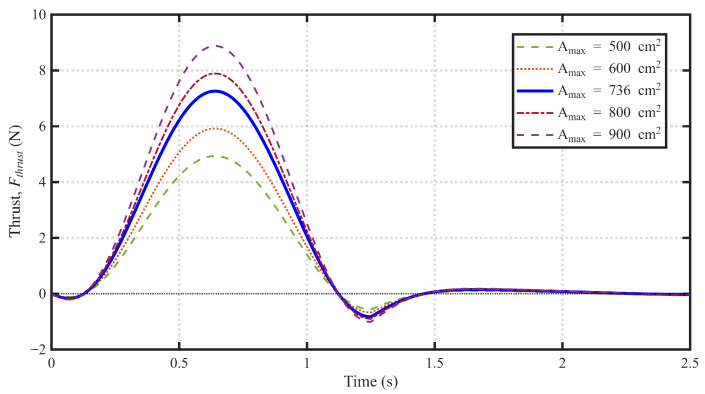
Estimated thrust for different maximum effective areas.

Figure 7 illustrates the simulated instantaneous thrust $F_{\mathrm{thrust}}\left(t\right)$ generated over a single flapping cycle based on this model. The plot compares the $F_{\mathrm{thrust}}\left(t\right)$ for several different values of maximum effective area $A_{\max}$. Our underwater robot uses an $A_{\max}$ of $736\;{\mathrm{cm}}^{2}$, which is predicted to generate a peak thrust of approximately 7.3 N.

## 4. Experiment

### 4.1. Static Thrust Performance

**Figure 8:**
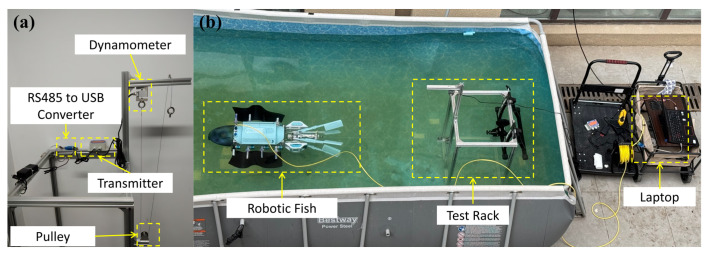
Robot thrust test experiment: (**a**) Thrust test device. (**b**) Schematic diagram of the experimental scene.

To investigate the propulsion characteristics of the two bionic propulsors, we designed and constructed an underwater static thrust testing platform. As shown in Figure 8a, the platform employs a dynamometer sensor as the thrust measurement unit, with signals transmitted to the host computer software via an RS485-to-USB converter. In the experimental scene illustrated in Figure 8b, the robot is submerged in water and secured to the test frame with a steel wire rope.

**Figure 9:**
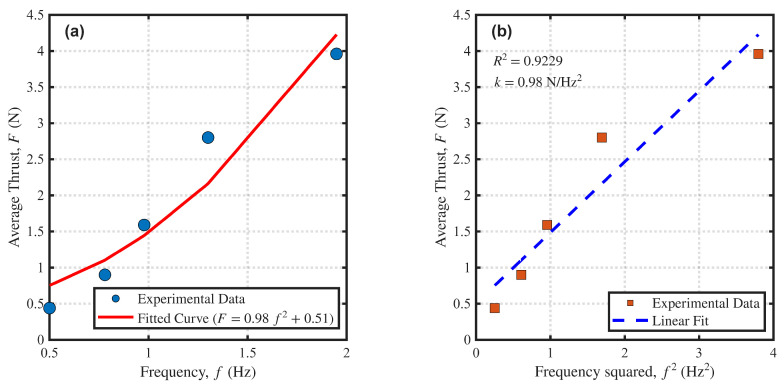
(**a**) Relationship between the static thrust of the undulating fin and oscillation frequency. (**b**) Relationship between the static thrust of the undulating fin and the square of oscillation frequency.

The thrust of the undulating fins was tested at oscillation frequencies of 0.500 Hz, 0.780 Hz, 0.975 Hz, 1.300 Hz, and 1.949 Hz. The experimental results are shown in Figure 9. Figure 9a shows the non-linear relationship between thrust and frequency. Through a second-order polynomial fit, we obtained the relationship $F=0.98f^{2}+0.51$. To more intuitively verify the theoretical model, Figure 9b plots the relationship between thrust and the square of the frequency. The data points show a strong linear trend, with a linear regression goodness-of-fit $R^{2}$ of 0.92 and a slope *k* of approximately 0.98. This result clearly confirms that the undulating fin static thrust is roughly proportional to the square of the oscillation frequency, which aligns with the theoretical derivation in Equation (5). Meanwhile, the experimental results align with observations and analyses of live fish, indicating that the propulsor designed in this study exhibits strong biomimetic properties and confirming the feasibility of the undulating fin propulsion mode [26,27].

**Figure 10:**
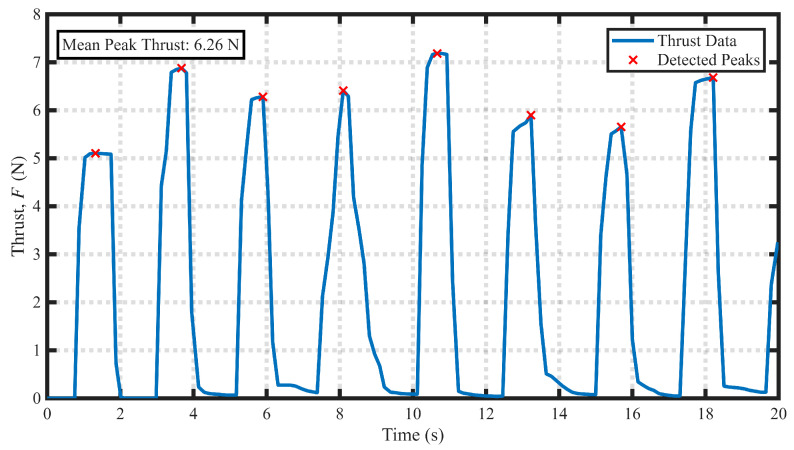
Relationship between the static thrust of the water-flapping tentacle and time variation.

The tail water-flapping tentacle operates in a single reciprocating motion mode. Therefore, our experimental objective was to investigate the thrust characteristics of this propulsor over time. The experimental results in Figure 10 clearly reveal that the thrust generated by this propulsor exhibits an intermittent burst pattern. After each power stroke, the thrust rapidly drops to near zero. By averaging the thrust peaks, we determined the mean peak thrust to be 6.26 N, which is in reasonable agreement with the 7.3 N peak thrust predicted by our simplified model in Section 3.2.

### 4.2. Dynamic Hydrodynamic Performance Analysis

While static tests provide baseline force data, they do not reflect the complex hydrodynamics of self-propulsion. To evaluate the robot’s actual swimming performance and the efficiency of the hybrid actuation, we conducted free-swimming experiments using a high-precision motion capture system by connecting the marker to the robot through a rigid rod.

#### 4.2.1. Drag Coefficient Identification

To accurately calculate the dynamic thrust during swimming, the hydrodynamic drag characteristics of the robot must first be identified. We performed coast-down tests where the robot was accelerated to a high speed and then allowed to decelerate naturally. Based on the equation of motion $m_{h}\dot{v}=-F_{\mathrm{drag}}=-\frac{1}{2}\rho C_{d}A_{r}v^{2}$, we fitted the deceleration curve using the motion capture data. The regression analysis yields a dimensionless drag coefficient of approximately $C_{d}=1.67$, which characterizes the hydrodynamic efficiency of the robot’s shape.

This experimentally determined coefficient allows us to decouple the thrust force from the net acceleration during forward swimming using the following relation:

(12) $$F_{\mathrm{thrust}}\left(t\right)=m_{h}\cdot a\left(t\right)+\frac{1}{2}\rho C_{d}A_{r}v{\left(t\right)}^{2}$$

where $m_{h}$ is the total hydrodynamic mass of the robot (15.4 kg), and $A_{r}$ is the frontal reference area of 0.062 m^2^.

#### 4.2.2. Multimodal Performance Comparison

We tested the robot under 7 operating conditions to compare the efficacy of the hybrid drive against independent propulsion modes:

1. Fin only: low (0.5 Hz), medium (0.65 Hz), and high (0.975 Hz) frequencies.
2. Tentacle only: standard bursting mode.
3. Hybrid mode: combining tentacles with the three fin frequencies.

**Figure 11:**
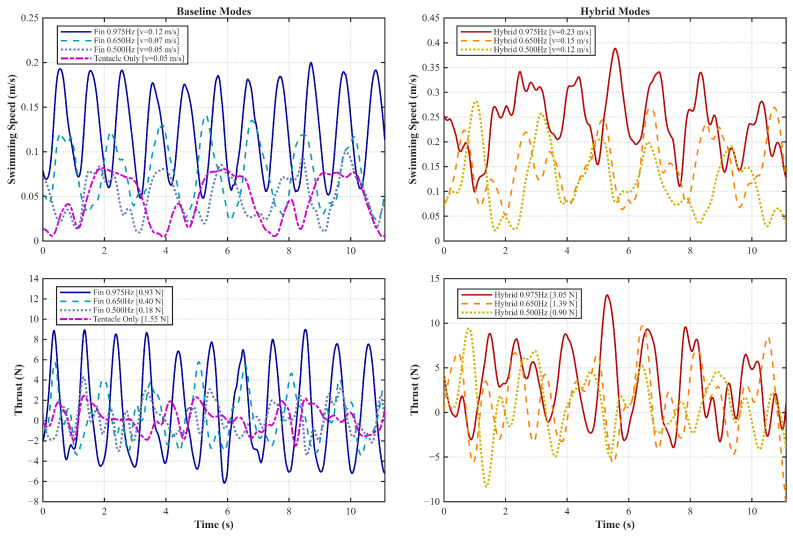
Detailed hydrodynamic performance comparison across 7 operating modes. Left column: baseline modes (Fin-only and Tentacle-only). Right column: hybrid modes (Fin + Tentacle).

Figure 11 presents the detailed time-domain response of swimming speed and generated thrust. It is noted that the plotted thrust represents the total dynamic force, incorporating both hydrodynamic drag and inertial effects. Consequently, negative thrust values are observed during the deceleration phases, reflecting the drag experienced by the propulsors during their recovery strokes. The fin-only mode produces continuous, oscillatory thrust, resulting in smooth velocity profiles. The tentacle-only mode generates high-impact thrust bursts but suffers from significant speed decay during the recovery stroke. Note that the thrust value of 1.55 N reported for the tentacle-only mode represents the mean peak thrust during active strokes, rather than the time-averaged value, which explains its high magnitude despite the lower average swimming speed. Crucially, the hybrid mode demonstrates the significant advantage of coupling these mechanisms. At the highest test frequency of 0.975 Hz, the hybrid activation increases the average swimming speed from 0.12 m/s to 0.23 m/s, an increase of approximately 92%. Furthermore, the average dynamic thrust in the hybrid mode reaches 3.05 N, far exceeding the sum of the independent components. This suggests a synergistic effect: the continuous propulsion from the undulating fins mitigates the speed loss during the tentacle’s recovery stroke, allowing the robot to maintain higher momentum and fully capitalize on the high-power burst of the flapping mechanism.

**Figure 12:**
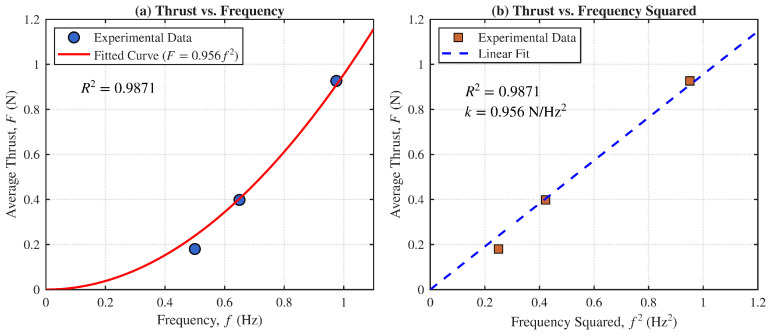
Dynamic thrust characterization of the undulating fin based on motion capture data: (**a**) Relationship between average thrust and frequency. (**b**) Relationship between average thrust and the square of the frequency.

Further analysis was conducted to characterize the relationship between the undulating fin’s oscillation frequency and the resulting dynamic mean thrust during free-swimming. As illustrated in Figure 12, the experimental data points derived from the mean values of the calculated dynamic thrust exhibit a strong quadratic correlation with the oscillation frequency. By fitting the data to the theoretical scaling law $F=kf^{2}$, we obtained a thrust factor $k=0.956\;{N/\mathrm{Hz}}^{2}$. The high coefficient of determination $R^{2}=0.9871$ indicates that the thrust generated by the undulating fins in a self-propelled state follows the hydrodynamic prediction described in Equation (5).

### 4.3. Efficiency and Cost of Transport Analysis

To quantitatively evaluate the energetic benefits of the hybrid actuation strategy, we analyzed the Propulsive Efficiency η and Cost of Transport (CoT) across different locomotion modes.

#### 4.3.1. Power Estimation and Metrics

Since onboard power logging was not available, the input power $P_{\mathrm{in}}$ was estimated based on the operating voltage and dynamic current models of the actuators. The undulating fins are driven by eight servos operating at 6.0 V, while the flapping tentacle mechanism is driven by a DC pusher at 12.0 V. The total input power is calculated as follows:

(13) $$P_{\mathrm{in}}=\sum_{i=1}^{8}\left(U_{\mathrm{servo}}\cdot I_{\mathrm{servo},i}\right)+\left(U_{\mathrm{pusher}}\cdot I_{\mathrm{pusher}}\right)$$

The useful output power $P_{\mathrm{out}}$ is derived from the hydrodynamic work performed against drag:

(14) $$P_{\mathrm{out}}=\bar{T}\cdot \bar{v}$$

where $\bar{T}$ is the average thrust and $\bar{v}$ is the average swimming speed.

Two key metrics were evaluated:

- Propulsive Efficiency (η): This represents the conversion rate of electrical energy to hydrodynamic work.
  (15) $$\eta =\frac{P_{\mathrm{out}}}{P_{\mathrm{in}}}\times 100\%$$
- Cost of Transport (CoT): This parameter measures the energy efficiency of transporting the robot’s mass over a unit distance. A lower CoT indicates better energy economy [28].
  (16) $$\mathrm{CoT}=\frac{P_{\mathrm{in}}}{mg\bar{v}}$$
  where *m* represents the physical mass of the robot.

#### 4.3.2. Comparative Results

Table 2 summarizes the estimated performance metrics. While the absolute propulsive efficiency is constrained by the thermal and frictional losses inherent in commercial servo actuators, the relative improvement offered by the hybrid mode is substantial.

The hybrid 0.975 Hz mode achieved the highest propulsive efficiency of 1.15%, which is approximately 4.6 times higher than the Fin-only mode of 0.25% at the same frequency. This indicates that although the hybrid mode consumes more instantaneous power ($P_{\mathrm{in}}\approx 61.89$ W vs. 45.09 W), the resulting surge in thrust and speed generates significantly more useful hydrodynamic work.

**Figure 13:**
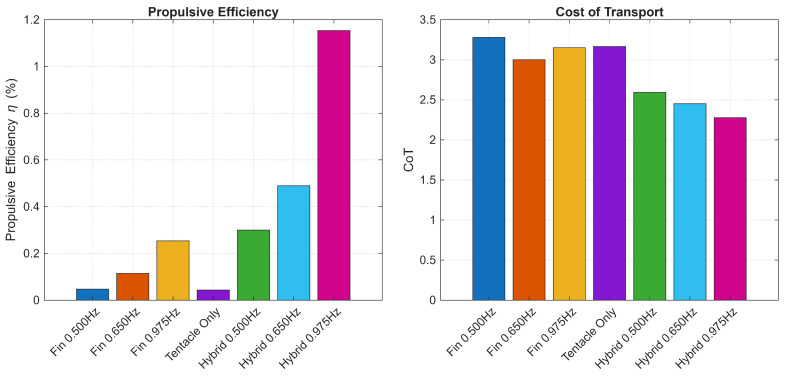
Efficiency performance metrics comparison.

The CoT analysis demonstrates that the hybrid robot is not only faster but also more economical per meter traveled. As shown in Figure 13, the CoT decreases from 3.15 to 2.28 at 0.975 Hz. The result that adding a high-power bursting mechanism reduces the cost of transport validates the hybrid design. By engaging the tentacles, the robot overcomes the drag threshold more effectively, allowing the velocity term $\bar{v}$ in the denominator of the CoT equation to increase faster than the power consumption $P_{\mathrm{in}}$ in the numerator.

To put these values in perspective, we compare them with the state-of-the-art. Bujard et al. [29] demonstrated a resonant squid-inspired robot that achieves a dimensionless CoT as low as 0.09, matching the efficiency of biological swimmers. Our robot’s minimum CoT of 2.28 is higher, which is expected given our use of standard commercial servo actuators and a rigid transmission system designed for multimodal versatility rather than resonance-based cruising. While our current energy efficiency does not yet match the biological optimum achieved by resonant soft robots, the hybrid drive strategy successfully reduces the CoT by 27.6% compared to the fin-only mode, proving the effectiveness of the proposed mechanism.

### 4.4. Maneuverability Verification

#### 4.4.1. Turning Test

To achieve high maneuverability in confined spaces, the robot employs a differential drive strategy utilizing the sinusoidal undulating fins. Unlike forward propulsion, where both fins undulate symmetrically, the turning maneuver is executed by generating counter-propagating waves on the opposing fins. Specifically, to execute a turn, the outer fin undulates from head to tail to generate forward thrust, while the inner fin undulates from tail to head to generate reverse thrust. During this process, the water-flapping tentacles remain in a folded, passive state to avoid hydrodynamic interference with the yaw motion.

To strictly quantify the turning performance and address the kinematics of the vehicle, free-swimming experiments were conducted using a high-precision motion capture system. The robot’s centroid trajectory and heading angle were tracked in real-time under three distinct fin oscillation frequencies.

**Figure 14:**
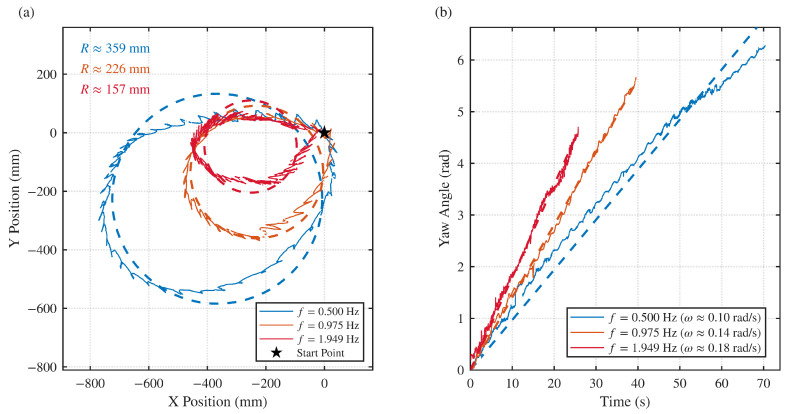
Quantitative turning performance analysis based on motion capture data: (**a**) Spatial trajectories of the robot centroid. (**b**) Time evolution of the yaw angle.

Figure 14a illustrates the spatial trajectories of the robot. A clear inverse relationship is observed between the oscillation frequency and the turning radius *R*. At a low frequency of f=0.500 Hz, the turning radius is approximately R≈359 mm. As the frequency increases to f=1.949 Hz, the radius significantly decreases to R≈157 mm, indicating that higher frequency undulations generate larger differential moments, resulting in sharper turns. This turning performance compares favorably with existing cephalopod-inspired robots. For instance, Wang et al. [30] reported a turning radius of approximately 0.5 body lengths (BL) for a soft robot utilizing a thrust-vectoring nozzle. In comparison, our robot, with a body length of 1.2 m, achieves a minimum turning radius of 157 mm, corresponding to approximately 0.13 BL. This indicates that the differential undulation of lateral fins provides superior yaw control authority compared to single-nozzle thrust vectoring, enabling agile maneuvering in confined underwater environments.

Figure 14b depicts the time-domain response of the robot’s yaw angle. The linearity of the curves suggests a stable turning rate, where the slope represents the angular velocity ω. Consistent with the trajectory data, the angular velocity is positively correlated with the oscillation frequency. The mean angular velocity increases from ω≈0.10 rad/s at 0.500 Hz to ω≈0.18 rad/s at 1.949 Hz. These results confirm that the robot can achieve agile, small-radius turning maneuvers solely through the differential control of the undulating fins, without reliance on the bursting propulsion of the tentacles.

#### 4.4.2. Diving and Surfacing Test

**Figure 15:**
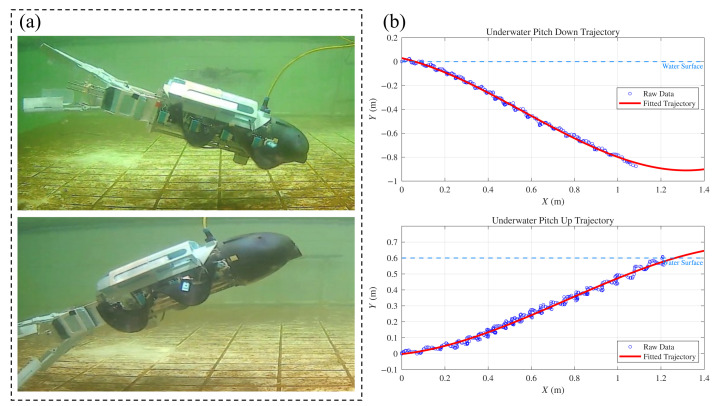
Experimental verification of diving and surfacing test: (**a**) Snapshots of the robot executing diving and surfacing motions in the test pool. (**b**) Measured spatial trajectories showing the pitch-down and pitch-up paths.

**Figure 16:**
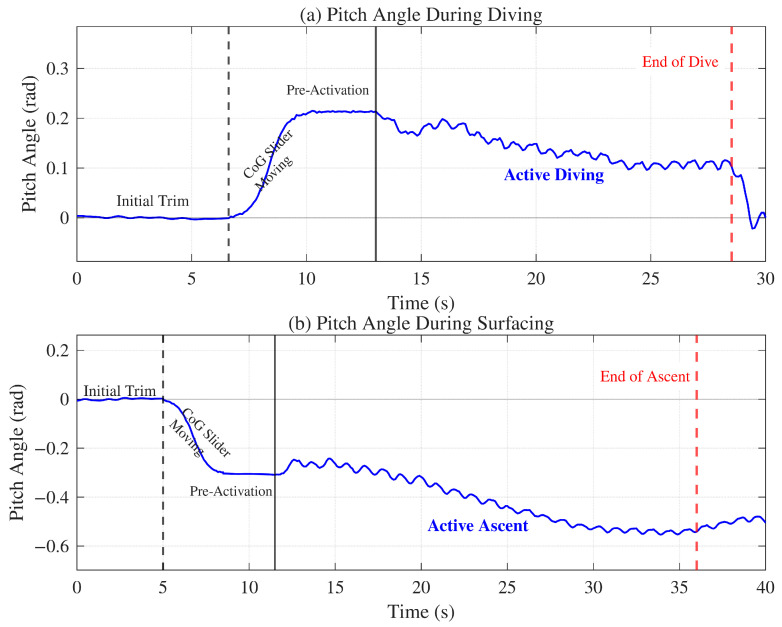
Time evolution of the pitch angle during diving and surfacing maneuvers: (**a**) Pitch angle response during the diving process. (**b**) Pitch angle response during the surfacing process.

The diving and surfacing capabilities of the robot rely on a coupled control strategy integrating the CoG adjustment module and the undulating fins. The spatial trajectory was tracked using external cameras, as shown in Figure 15, while the pitch angle was recorded in real-time using the onboard IMU, as illustrated in Figure 16. To initiate a dive, the stepper motor drives the CoG forward, generating a nose-down pitching moment. Consequently, the robot naturally tilts until it reaches a stable pitch-down angle due to hydrostatic restoring moments. The undulating fins are activated to generate forward thrust. Under the established pitch angle, the hydrodynamic forces propel the robot along a descending trajectory. Similarly, for the surfacing maneuver, the CoG is shifted to the rear, creating a pitch-up moment. After the robot stabilizes at the target pitch angle, the fins drive the robot to ascend. These experiments quantitatively confirm that the CoG adjustment mechanism effectively modulates the robot’s attitude to achieve depth control.

#### 4.4.3. Open-Water Swimming

**Figure 17:**
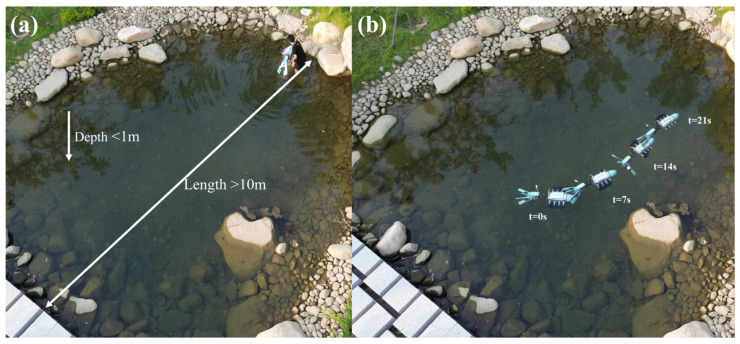
Outdoor Environment Experiment: (**a**) Experimental Environment. (**b**) Outdoor Experiment Results.

To evaluate the locomotion of the robot in real-world environments and the adaptability of its water-flapping tentacles in confined spaces, experiments were conducted in a small artificial lake, and the entire process was recorded using a drone. The artificial lake, shown in Figure 17a, has a depth of approximately 1 m at its center, implying that when the water-flapping tentacles were fully extended, they were likely to strike the stones on the lakebed. Owing to the adaptive capability of the water-flapping tentacles, the robot successfully completed its movement in the lake without any damage to its mechanical structure, as tracked in Figure 17b.

## 5. Discussion

While the hybrid-actuated cephalopod-inspired robot demonstrates promising maneuverability and adaptability, several limitations in its current design warrant discussion.

First, regarding spatial maneuverability, the depth control mechanism relies on a gliding strategy. Since the robot is designed with positive buoyancy and lacks independent vertical thrusters, diving and surfacing are achieved by coupling pitch attitude with forward propulsion. This implies that the robot cannot execute vertical heave motions while hovering at zero speed; a minimum forward velocity is required to generate the necessary hydrodynamic lift for depth changes.

Second, concerning environmental robustness, the experiments reported in this study were conducted in controlled, quiescent water environments. Although the bio-inspired streamlined fuselage and the low center of gravity provide inherent stability, the large projected areas of the undulating fins and flapping tentacles may be susceptible to external disturbances. In environments with strong turbulence or significant wave surges, these flexible structures might experience uncommanded deformation or drag, potentially affecting trajectory tracking accuracy. Future iterations will require closed-loop control algorithms specifically tuned to compensate for such environmental disturbances.

Finally, the current prototype relies on a tethered communication system for remote control, which limits the operational radius. Future work will focus on integrating fully onboard perception and decision-making modules to achieve autonomous operation in more complex, dynamic ocean environments.

## 6. Conclusions

This paper proposed and developed a new type of underwater bionic robot. By studying the multimodal swimming mechanisms of cephalopods, specifically the coordination of fins and tentacles, a bionic robot with integrated propulsion was developed. Its innovation lies in adopting a composite propulsion mode combining sinusoidal undulating fins and water-flapping tentacles to replace traditional propellers. The system also relies on the CoG adjustment module to achieve stable pitch control for ascent and descent. Through a large number of experimental verifications, the movement laws of the two bionic propulsors were explored, and the multimodal motion performance of the whole robot was verified in different scenarios. Propulsor experiments confirmed the theoretical models, showing that the undulating fin thrust scales quadratically with frequency, and that the adaptive tentacles produce intermittent thrust. Vehicle performance tests demonstrated high maneuverability by enabling pitch control for diving and surfacing maneuvers and achieving a turning radius of approximately 15 cm. These tests also showed excellent environmental adaptability, as the adaptive tentacle structure allowed it to successfully navigate a shallow lake with a depth of less than 1 m and endure contact with underwater obstacles without damage. In conclusion, the combination of high maneuverability and robust environmental adaptability demonstrated by this robotic platform validates its significant potential as a novel tool for underwater observation tasks in specialized and complex environments.

## Figures and Tables

**Table 1:** Specifications of the robot.

Item	Specifications
Dimensions	1200 mm × 450 mm × 310 mm
Weight	11.84 kg
MCU	STM32F103RCT6
IMU	WT1-IMU
Servos	63 kgf·cm × 8
Electric drive pusher	12V 60 mm/s 90 N
Battery	4 S 5200 mAh

**Table 2:** Performance comparison of propulsion modes.

Mode	Freq	Speed	Thrust	Power ($P_{\mathrm{in}}$)	Eff (η)	CoT
	(Hz)	(m/s)	(N)	(W)	(%)	(-)
Tentacle only	-	0.046	0.16	16.80	0.04	3.17
Fin only	0.500	0.051	0.18	19.37	0.05	3.28
	0.650	0.075	0.40	26.06	0.11	3.00
	0.975	0.123	0.93	45.09	0.25	3.15
Hybrid	0.500	0.120	0.90	36.17	0.30	2.59
	0.650	0.151	1.39	42.86	0.49	2.45
	0.975	0.234	3.05	61.89	1.15	2.28

## Data Availability

The data presented in this study are available upon request from the corresponding author.
